# Evaluation of a novel virtual screening strategy using receptor decoy binding sites

**DOI:** 10.1186/s12952-016-0058-8

**Published:** 2016-08-23

**Authors:** Hershna Patel, Andreas Kukol

**Affiliations:** Department of Biological and Environmental Sciences, University of Hertfordshire, College Lane, Hatfield, AL10 9AB UK

**Keywords:** Virtual screening, Docking, Receptor-decoy, Enrichment, Adjusted ranking, Autodock Vina

## Abstract

Virtual screening is used in biomedical research to predict the binding affinity of a large set of small organic molecules to protein receptor targets. This report shows the development and evaluation of a novel yet straightforward attempt to improve this ranking in receptor-based molecular docking using a receptor-decoy strategy. This strategy includes defining a decoy binding site on the receptor and adjusting the ranking of the true binding-site virtual screen based on the decoy-site screen. The results show that by docking against a receptor-decoy site with Autodock Vina, improved Receiver Operator Characteristic Enrichment (ROCE) was achieved for 5 out of fifteen receptor targets investigated, when up to 15 % of a decoy site rank list was considered. No improved enrichment was seen for 7 targets, while for 3 targets the ROCE was reduced. The extent to which this strategy can effectively improve ligand prediction is dependent on the target receptor investigated.

## Background

Virtual screening is a widely used technique in the field of medicinal chemistry to identify lead compounds from a diverse library that can bind to a receptor. The receptor based virtual screening approach involves a process called molecular docking which employs an algorithm that docks each molecule from a library into the binding site in order to predict a binding energy or a binding score [1]. In recent years, a number of successful virtual screening based studies have been conducted as described for example in the recent review by Lavecchia et al. [2]. Although docking provides an efficient and cost effective way to assess interactions between molecules such as proteins and ligands on a large-scale, the accuracy, as defined by the ability to predict strong binding ligands, is limited. This is largely due to the limitation of scoring functions used in the software to calculate binding energies, and therefore their ability to identify true positives from a database composed of known ligands and decoys that is typically used in evaluations of virtual screening [3, 4]. The accuracy of the screening method can be assessed quantitatively through calculation of the robust metric known as Receiver Operator Characteristic Enrichment (ROCE) [5]. An ROCE factor is obtained as the true positive rate divided by the false positive rate, thus ROCE factors much larger than 1.0 are desirable to establish that the docking algorithm can distinguish active compounds from decoys.

Several software for molecular docking are available [6] and have been evaluated [7, 8]. Furthermore, methods to increase the accuracy of virtual screening have been suggested, for example considering receptor flexibility to reduce the numbers of false positive molecules [9], consensus docking to predict correct binding pose [10], and a consensus virtual screening method that combined the rank lists of ligands from different algorithms [11]. However, these improved methods can still result in a low number of correct predictions for some receptors [11]. In the work described here the novel strategy of using receptor decoy sites was developed and evaluated for the first time together with the docking software AutoDock Vina [12]. This involved performing virtual screening against a non-binding (receptor-decoy) site on the same protein target, and developing a way to re-rank the screening results, thus enabling a comparison of ROCE factors before and after the application of receptor-decoy screening in order to evaluate the novel strategy.

## Methods

Ligand and decoy sets for fifteen target proteins were downloaded from the Database of Useful Decoys [3]. The complexes were selected from several different protein categories in the database such as hormone receptors, kinases, proteases and other enzymes to represent a wide range of targets, including 10 targets which had previously been evaluated [11]. Virtual screening for all fifteen targets was performed using Autodock Vina version 1.1.1 with the default parameters [12]. The FTMap binding site prediction server [13] was used to help define the decoy site for docking. The FTMap server identifies binding hot-spots by computational solvent mapping whereby 16 different molecular probes are docked onto the protein surface to locate favorable binding regions [13]. The decoy site was chosen based on the following criteria: 1) contains no binding hotspot predicted by FTMap, 2) it appears structurally different to the actual binding site and 3) it does not form an obvious binding cavity but is at a flat region on the exterior surface of the protein. The search space for docking was defined via a grid box manually specified with Autodock Tools [14] around the binding or decoy site. A grid spacing of 0.375 Å was used to determine the box dimensions. The box dimensions remained the same for binding site and decoy site docking. Adjusted rank lists were generated from the binding site list by considering molecules that were in the top 10 %, 15 %, 20 %, 30 % and 50 % of the decoy site list, and adjusting the rank of the binding site list using the following formula:

$$ Adjusted\; rank=\left( Binding\; site\; rank- Decoy\; site\; rank\right)+ Total\; no. of\; ligands\; in\; list $$

The fraction of decoy-site docking results was varied in order to find a cut-off where maximum enrichment is achieved. The numbers of active ligands in the database were then used to calculate the ROC Enrichment (ROCE) factors at 1 % and 2 % of the number of molecules. The ROCE_x%_ was calculated as the fraction of true positives divided by the fraction of false positives at x% of the ligand/decoy database according to the equation:

$$ ROC{E}_{x\%}=\frac{f_{actives}}{1-\frac{\left({N}_{decoys}-{N}_{inactives}\right)}{N_{decoys}}} $$

Where *f*_*actives*_ = (number of actives at x%) / (number of all actives),

*N*_*decoys*_ = the total number of inactive decoys,

*N*_*inactives*_ = the number of decoys chosen at x% of the ligand/decoy database.

Binding site and decoy sites were analysed post-docking with the KVFinder Cavity Detection PyMol Plugin [15] to provide a quantitative description of the two sites. The software enables comparison and characterisation of protein binding sites by the number, area and volume of cavities in a specified search space. The default parameters were used for all fifteen targets which included a probe in size of 1.4 Å, probe out size of 4.0 Å and a step size of 0.6 Å. The minimum cavity volume was set at 5.0 Å. The binding site search space was set around the position of the actual ligand molecule obtained from the Protein Data Bank, and the decoy site search space was set using a docked molecule from the decoy site screening.

## Results and discussion

**Fig. 1:**
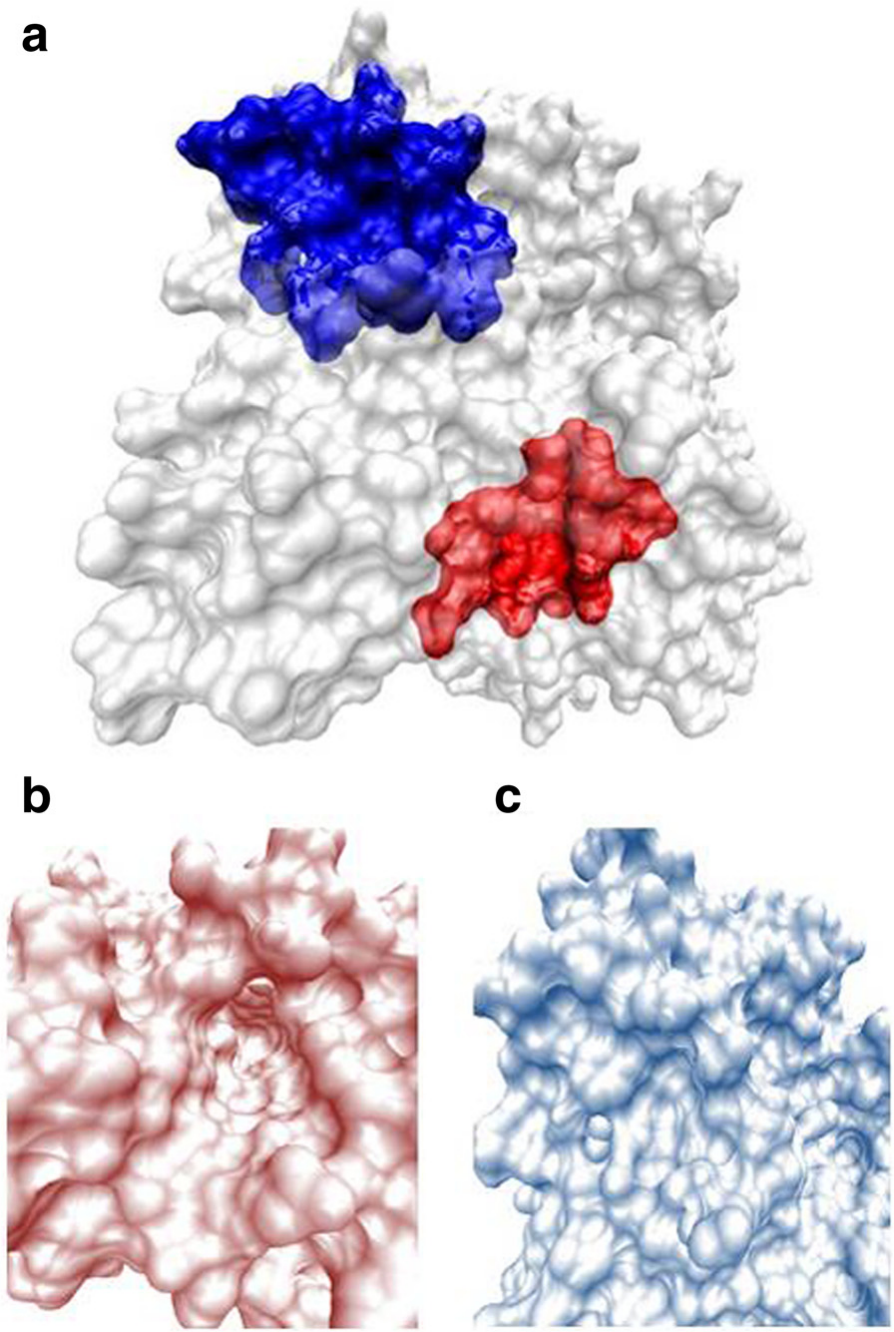
**a** Acetycholine esterase (Ache) receptor with binding site shown in red and decoy site in blue. **b** Detailed view of Ache binding site. **c** Detailed view of Ache decoy site

High predicted binding affinities between a ligand and a receptor may not always correspond with the best binding molecules for the target site investigated [6, 16]. In virtual screening this is reflected by low enrichment factors which indicate that many of the highest ranked molecules may be false positive predictions [5]. In this study, the level of Receiver Operator Characteristic Enrichment (ROCE) was determined at fractions of 1 % and 2 % of the dataset of ligand/decoy molecules obtained from the Database of useful Decoys [3]. Docking against a non-binding ‘decoy’ site on the same receptor (Fig. 1) was carried out using the software Autodock Vina that lead to a ranking of molecules different from the ranking for the true binding site. The predicted binding energies among top molecules for the decoy site were less negative than for binding sites, indicating a lower degree of binding to the decoy site. The ranking for the true binding site was adjusted by considering a varied fraction of the rank list produced from the decoy site from 0 % (no correction) to 50 % (Tables 1 and 2).

**Table 1:** ROCE at 1 % of the binding site list considering top x% of the decoy site list

Top % of decoy site list						
Target	0	10	15	20	30	50
Comt	33.4^a^	39.0	39.0	39.0	39.0	39.0
AchE	1.0^a^	1.0	3.0	3.0	2.0	3.03
CDK2	14.3^a^	23.6	29.7	23.6	29.7	14.3
HIVrt	9.4^a^	13.1	13.1	9.0	13.1	18.0
Pparg	58.0^a^	99.0	84.0	84.0	84.0	84.0
FGFR1	0.0	0.0	0.9	0.9	0.9	0.9
InhA	14.6	5.3	5.3	5.3	2.5	0.0
PR	0.0	0.0	0.0	0.0	0.0	4.0
RXRa	212.4	212.4	212.4	88.5	47.2	88.5
VEGFr2	10.2	2.9	2.9	2.9	1.4	1.4
MR	178.3	178.3	71.3	71.3	71.3	71.3
Hsp90	0.0	0.0	0.0	0.0	0.0	0.0
AmpC	0.0	0.0	0.0	0.0	0.0	0.0
Trypsin	5.0	5.0	5.0	5.0	5.0	5.0
Parp	7.1	7.1	7.1	7.1	7.1	11.9

^a^Results taken from ref [11]

**Table 2:** ROCE at 2 % of the binding site list considering top x% of the decoy site list

Top % of decoy site list						
Target	0	10	15	20	30	50
Comt	10.4^a^	11.2	11.2	11.2	11.2	11.2
AchE	1.5^a^	2.0	2.0	1.5	2.0	1.5
CDK2	13.3^a^	15.7	15.7	15.7	11.9	5.8
HIVrt	8.8^a^	9.0	9.0	7.2	7.2	11.0
Pparg	35.8^a^	58.0	54.0	58.0	50.0	44.0
FGFR1	0.6	0.4	0.4	0.4	0.4	0.4
InhA	12.4	3.1	3.1	2.5	1.2	0.0
PR	0.0	0.0	0.0	0.0	1.9	4.0
RXRa	70.8	97.4	70.8	40.5	17.7	23.6
VEGFr2	5.3	1.4	2.1	2.1	2.9	1.4
MR	62.4	42.8	29.7	20.4	20.4	3.6
Hsp90	0.0	0.0	0.0	0.0	0.0	4.5
AmpC	0.0	0.0	0.0	0.0	0.0	0.0
Trypsin	5.0	5.0	5.0	5.0	5.0	5.0
Parp	5.1	3.2	7.1	7.1	7.1	9.4

^a^Results taken from ref [11]

The results show a considerable variation between the fifteen targets investigated confirming the general consensus that virtual screening accuracy is highly dependent on the target (Tables 1 and 2). Overall, the majority of targets did not show any improvement in enrichment at the top 1 % or 2 % of the list after applying the receptor decoy method. Five targets (Comt, Ache, CDK2, HIVrt and Pparg) show improved ROCE factors compared to those obtained in the previous study [11], (see footnotes in Tables 1 and 2) when considering at least the top 15 % of the decoy site list. Beyond 15 % the enrichment for all targets (except HIVrt and Parp) either remained constant or dropped to a lower value.

The rationale behind the receptor decoy strategy was that the number of false positive binders could be reduced by determining molecules, which have a tendency to bind non-specifically to molecular surfaces that are different to the binding site. As a result a higher number of active ligands would remain after adjusting the rank list for the true binding site with the rank list for the decoy site. However, the results show that this approach is unlikely to help in the identification and selection of molecules for experimental testing as a higher number of true positives were recalled for only 5 out of 15 targets. The extent of enrichment achieved for the top 1 % and 2 % differed for all targets due to properties that determine the binding interactions between amino acid residues of the target and the ligand-decoy dataset used for docking. The optimum cut-off for maximum enrichment at the top 1 % of a binding site list was obtained when considering 15 % of the decoy list (Table 1), and 10 % for the top 2 % of the binding site list (Table 2). This shows that the ranking of molecules with regards to binding to the decoy sites is meaningless for lower ranks.

The largest improvement in enrichment was achieved with the targets CDK2 and Pparg. For the targets PR, Hsp90 and ampC the ROCE at 1 % and 2 % remained at zero until considering at least 30 % of molecules in the decoy list, indicating that true and false ligands cannot be distinguished by the Autodock Vina docking algorithm. Cavity analyses of the binding site and decoy site (Table 3) using the software KVFinder [15] shows that the total number, volume and area of the cavities found in the decoy site were smaller in comparison to the binding site for all targets except HIVrt and trypsin. This confirms that the shapes of the 2 sites are very different, although this did not prevent false positive molecules binding with high affinity.

**Table 3:** Cavity analysis of binding sites and decoy sites for all targets using KVFinder [15]

	Binding site Cavities			Decoy site cavities		
Receptor	Number	Total Volume (Å^3^)	Total Area (Å^2^)	Number	Total Volume (Å^3^)	Total Area (Å^2^)
Comt	1	29.8	45.7	1	12.3	20.3
AchE	3	249.3	333.8	2	85.5	124.9
CDK2	4	134.0	178.3	1	10.6	15.8
HIVrt	5	92.1	138.7	1	241.3	240.1
Pparg	2	394.4	414.8	1	8.6	14.4
FGFR1	2	49.0	70.2	1	21.0	30.6
InhA	2	1119.7	834.8	1	6.0	10.1
PR	2	21.4	35.0	1	18.1	28.4
RXRa	1	57.5	72.0	1	21.0	30.2
Vegfr2	5	129.3	193.0	4	117.4	168.0
MR	2	54.4	78.1	1	23.5	35.6
Hsp90	4	166.8	233.7	2	30.0	46.1
ampC	3	100.8	121.3	1	5.8	9.7
trypsin	1	9.7	14.8	3	79.9	121.8
Parp	1	538.3	482.4	1	9.9	16.6

The targets Inha, MR and VEGFr2 show a significant decrease in ROCE indicating this strategy makes the retrieval of active ligands in the top ranks worse for these targets. The actual binding site for VEGFr2 appears to be non-specific, open and flat, therefore binds molecules which also bind easily to the decoy site, resulting in a high proportion of active molecules at the top of the decoy list. However, the Inha binding site is a small, deep pocket with a total cavity area of 838.4 Å^2^ which appears not to be easily surface accessible, so it is expected that this receptor only binds ligands which are complementary in shape. Although, this was not seen as a higher number of active ligands were found in the top 1 % of the decoy site list compared to the binding site list. Thus, when the re-ranking formula to generate the adjusted list is applied, the binding site list is re-ordered such that the active ligands do not appear in the top positions. This highlights the shortcoming, if applying this strategy to a virtual screening experiment where active molecules are not known, it cannot be guaranteed that any improved prediction accuracy will result.

## Conclusion

The novel development and evaluation of docking with a decoy binding site shows that improved prediction of active ligands could not be achieved in general. It should be noted that the ligand/decoy dataset used for this evaluation is especially challenging as decoys physico-chemical similar to ligands were chosen [3]. The choice of appropriate decoy binding sites is critical for the success of this method. Choosing an obviously unfavorable site, such as a flat molecular surface, reduces the docking scores overall and thus the potential to discriminate between ligands and decoys, while on the other hand the choice of an alternative binding cavity might cause a novel mode of specific binding that does not help to eliminate the false postives for the true binding site. The question, how to define a decoy binding site, such that false positive predictions for the real binding site are removed must remain open and is put forward to the academic community. Further work addressing the re-ranking of predicted ligands may also lead to improvements.

## Abbreviations

AchE: Acetycholine Esterase
ampC: Ampicillin Class C
CDK2: Cyclin Dependent Kinase 2
Comt: Catechol O-methyltransferase
FGFR1: Fibroblast Growth Factor Receptor Kinase
HIVrt: HIV Reverse Transcriptase
Hsp90: Heat Shock Protein 90
InhA: Enoyl ACP Reductase
MR: Mineralocorticoid Receptor
parp: Poly (ADP-ribose) Polymerase
Pparg: Peroxisome Proliferator Activated Receptor Gamma
PR: Progesterone Receptor
ROCE: Receiver Operator Characteristic Enrichment
RXRa: Retinoic X Receptor Alpha
VEGFr2: Vascular Endothelial Growth Factor Receptor Kinase
